# Severity of premenstrual symptoms among women with musculoskeletal pain: relation to vitamin D, calcium, and psychological symptoms

**DOI:** 10.25122/jml-2023-0050

**Published:** 2024-04

**Authors:** Khalid Abdul-Razzak, Eman Alshdaifat, Amer Sindiani, Mohammad Alkhatatbeh

**Affiliations:** 1 Department of Clinical Pharmacy, Faculty of Pharmacy, Jordan University of Science and Technology, Irbid, Jordan; 2 Department of Obstetrics and Gynecology, Faculty of Medicine, Yarmouk University, Irbid, Jordan; 3 Department of Obstetrics and Gynecology, Faculty of Medicine, Jordan University of Science and Technology, Irbid, Jordan

**Keywords:** premenstrual syndrome, musculoskeletal pain, anxiety, depression, calcium, vitamin D, BMI, body mass index, HADS, Hospital Anxiety and Depression Scale, MSP, musculoskeletal pain, PMDD, premenstrual dysphoric disorder, PMS, premenstrual syndrome

## Abstract

Premenstrual syndrome (PMS) has various symptoms that occur during the luteal phase of the menstrual cycle and subside after menstruation. Anxiety and depression are prevalent in women with PMS and may exacerbate the severity of PMS. Vitamin D and calcium deficiency may have a role in developing anxiety, depression, and musculoskeletal pain (MSP). The aim of this study was to evaluate selected premenstrual symptoms in relation to serum vitamin D levels, daily calcium consumption, and psychological symptoms among women with MSP. The study population consisted of 108 women with MSP and 108 healthy controls. Information about premenstrual symptoms and calcium consumption were collected. Psychological symptoms were assessed using the Hospital Anxiety and Depression Scale (HADS). Vitamin D was determined by electrochemiluminescence immunoassay. Women with MSP had lower serum vitamin D levels, lower daily calcium consumption, higher HADS scores for anxiety and depression, and higher frequency of severe premenstrual symptoms including fatigue, headache, irritability, mood swings, anxiety, depression, and social withdrawal compared to controls (*P* < 0.01). Abnormal HADS scores for anxiety and depression were associated with increased severity of premenstrual symptoms (*P* < 0.05). Deficient vitamin D and calcium consumption were associated with abnormal HADS scores for anxiety and depression (*P* < 0.05) and with increased severity of premenstrual headache, irritability, anxiety, and depression (*P* < 0.05). Low calcium consumption was associated with increased severity of premenstrual irritability, anxiety, depression, and social withdrawal (*P* < 0.05). The results suggest that vitamin D deficiency, low calcium consumption, psychological symptoms, and MSP could be interrelated and implicated in the etiology severe premenstrual symptoms. Further studies are necessary to assess whether vitamin D and calcium supplements can relieve MSP and premenstrual symptoms.

## INTRODUCTION

Premenstrual syndrome (PMS) is a common disorder among women in their reproductive age. Women with PMS suffer from physical, emotional, and behavioral symptoms that occur in the luteal phase of the menstrual cycle and subside after menstruation [1]. Common physical symptoms of PMS include headache, breast tenderness, fatigue, lack of energy, abdominal bloating, and peripheral edema, whereas psychological and behavioral symptoms include irritability, mood swings, social withdrawal, anxiety, and depression [2,3].

The emotional and behavioral symptoms of PMS can have a substantial negative effect on women’s daily activities and professional performance, as well as their social and work relationships [4]. In extreme cases, PMS may also lead to suicide [5]. PMS with extreme emotional symptoms is categorized as premenstrual dysphoric disorder (PMDD), which affects 3–8% of women with PMS [6].

The pathophysiology of PMS is not fully understood. Fluctuations in estrogen and progesterone, along with changes in neurotransmitters, particularly serotonin, during the menstrual cycle, may be important in the development of PMS [7–9]. Sex steroids and their receptors are abundant in the brain, where they regulate emotions and behaviors and modulate the secretion of serotonin, which is involved in the etiology of PMS [10] and in the manifestation of irritability, anger, and depressive symptoms [9]. Genetics and lack of micronutrients are also suggested to contribute to the etiology of PMS [11–13].

Psychological symptoms are common among women with severe PMS and are believed to exacerbate the severity of premenstrual symptoms [7,10]. Studies have found a significant association between psychological symptoms and the incidence and severity of dysmenorrhea and PMS [4,14,15]. Women with depressive symptoms are more likely to experience PMS and its most severe form, PMDD [13]. PMDD has significantly higher rates of occurrence in women with certain psychiatric disorders, including dysthymia, major depressive disorder, panic disorder, and generalized anxiety disorder, which correlates with painful menstruation, and higher scores on the neuroticism (emotional stability) scale [11,16].

Vitamin D has an important role in calcium homeostasis and the health of the brain and nervous system [17,18]. Chronic pain, including musculoskeletal pain (MSP), and weakness are symptoms often associated with vitamin D deficiency [19–21].

MSP is a major health issue, being common in all subgroups of the population [22,23]. It often coexists with anxiety and depression, and they can exacerbate each other. They share the same biological pathways and neurotransmitters and respond to similar treatments [24,25].

Previously, we found that low serum vitamin D level and low daily calcium consumption were associated with anxiety and depression symptoms among patients with MSP and psychiatric outpatients [26,27]. However, to the best of our knowledge, no previous study has assessed the prevalence and severity of premenstrual symptoms among women with MSP. Therefore, the aim of this study was to evaluate the frequency and severity of premenstrual symptoms in relation to vitamin D status, daily calcium consumption, and psychological symptoms among women with MSP.

## MATERIALS AND METHODS

### Study design

This study included 108 women with MSP and 108 age-matched healthy women with no MSP. Participants with MSP were recruited from physician referrals to physical therapy and neurology clinics, from the department of radiology at King Abdullah University Hospital, and by announcing the research project at the campus of Jordan University of Science and Technology, Irbid, Jordan. Participants with no MSP were recruited from women who were visiting various clinics for checkup, or patients’ family members as well as from the university campus.

To avoid possible confounders, we excluded women with chronic diseases such as chronic renal impairment and chronic liver disease, women who took vitamin D supplements in the previous 2 months, as well as women who were at menopause or pregnant. Inclusion criteria were age between 18 and 45 years, willingness to participate, and providing written informed consent.

### Data collection

Demographic information including age, marital status, and data about complaints of chronic MSP were obtained by self-reporting. Body mass index (BMI) was calculated in kg/m^2^.

### Assessment of premenstrual symptoms

The participants were requested to provide information about their recurrent experience of five psychological symptoms that occur during the premenstrual phase and subside with the onset of menstruation: irritability, feeling depressed, anxiety, mood swings, and social withdrawal [28]. In addition, information about three physical symptoms (breast tenderness, headache, and fatigue) were recorded. Symptom severity was categorized based on a premenstrual severity scale with 21 items, in which each item was rated with a score of 0 for none, 1 for mild, 2 for moderate, and 3 for severe. The total score was calculated by summing the 21 item scores. A score of ≤22 indicated mild symptoms, 23–36 indicated moderate symptoms, and ≥37 indicated severe symptoms.

### Assessment of daily calcium consumption

Information regarding dairy consumption including milk, yogurt, cheddar cheese, cream cheese, and labneh (a type of Arabian yogurt without whey) were collected. Dairy consumption was reported as 0, 1, 2, ≥3 servings per day, a serving being defined as 1 cup of milk or yogurt (300 mg calcium), 2 full tablespoons (2 oz.) of labneh (100 mg calcium), a 1-ounce piece of cheese (162 mg calcium), or a 1-ounce serving of cream cheese (20 mg calcium) [26,29]. For participants who were taking calcium supplements, the amount of supplemented calcium was added to the amount calculated from dairy consumption to determine the total daily calcium consumption.

### Psychosocial questionnaire

Self-reported symptoms of anxiety and depression were assessed using the Hospital Anxiety and Depression Scale (HADS), which is a simple and reliable tool commonly used to evaluate anxiety and depression disorders [30]. The HADS consists of seven questions measuring the level of anxiety and seven questions measuring the level of depression. Each question is graded on a four-point scale ranging from 0 (not at all) to 3 (very often). Accordingly, study participants were classified into three groups based on their total score, as follows: normal (score 0–7), borderline abnormal (score 8–10), and abnormal or clinical case of anxiety or depression (score 11–21).

### Biochemical measurements

Laboratory measurements were performed at the laboratories of King Abdullah University Hospital. Venous blood samples were obtained to measure 25-hydroxyvitamin D concentration in the serum using the electrochemiluminescence immunoassay (Roche Modular E170 Analyzer, Roche Diagnostics). Serum vitamin D levels of ≥30 ng/dl were considered normal, 29–20 ng/dl insufficient, and <20 ng/dl deficient.

### Statistical analysis

Statistical analyses were performed using SPSS v.21 (IBM Corp). Categorical variables were presented as frequency (%). Continuous variables were tested for normality and presented as mean ± s.d. or median (25^th^–75^th^ percentiles). Independent samples *t*-test or the Mann–Whitney *U*-test were used to determine differences in continuous variables between women with and without MSP. The chi-squared test was used to find associations between categorical variables. A *P* value of ≤0.05 was considered statistically significant.

## RESULTS

### Characteristics of the study sample

Data were obtained by interviewing 108 women with MSP (study group) and 108 healthy women without MSP (control group), aged between 18 and 45 years. The characteristics of the study population are presented in Table 1. The mean age of the study group and the control group was 27.69 ± 7.26 years and 27.78 ± 6.9 years, respectively; (*P* = 0.92). The majority of participants were single (63% of the study group vs. 48.1% of the control group; *P* = 0.04). Mean BMI was 24.91 ± 4.91 in the study group and 23.81 ± 4.11 in the control group (*P* = 0.08).

**Table 1:** Characteristics of the study population

Variables	Women with MSP (*n* = 108)	Women without MSP (*n* = 108)	*P* value
Age, years	27.69 ± 7.26	27.79 ± 6.86	0.92
BMI, kg/m^2^	24.91 ± 4.91	23.81 ± 4.11	0.08
**Marital status**			
Single	68 (63)	52 (48.1)	0.04^*^
Married	40 (37)	56 (51.9)	
Mean serum vitamin D, ng/dl	7.35 (4.0–12.58)	18.22 (8.97–29.55)	<0.001^*^
**Serum vitamin D**			
Normal (≥30 ng/dl)	5 (4.6)	26 (24.1)	<0.001^*^
Insufficient (29–20 ng/dl)	10 (9.3)	22 (20.4)	
Deficient (<20 ng/dl)	93 (86.1)	60 (55.6)	
Mean daily calcium consumption, mg	205.36 (81.89–358.79)	469.76 (304.47–694.16)	<0.001^*^
**Daily calcium consumption**			
<230 mg/day	56 (51.9)	16 (14.8)	<0.001^*^
230–450 mg/day	37 (34.3)	35 (32.4)	
>450 mg/day	15 (13.9)	57 (52.8)	
Mean HADS anxiety score (0–21)	10.51 ± 4.41	5.44 ± 3.55	<0.001^*^
**HADS anxiety score**			<0.001^*^
Normal (0–7)	28 (25.9)	87 (80.6)	
Borderline (8–10)	26 (24.1)	9 (8.3)	
Abnormal (11–21)	54 (50)	12 (11.1)	
Mean HADS depression score (0-21)	9.16 ± 3.62	4.83 ± 2.79	<0.001^*^
**HADS depression score**			<0.001^*^
Normal (0–7)	39 (36.1)	89 (82.4)	
Borderline (8–10)	30 (27.8)	15 (13.9)	
Abnormal (11–21)	39 (36.1)	4 (3.7)	

* Statistically significant. Data expressed as frequency (%). Mean values expressed as mean ± s.d.

### Differences in study variables between women with and without MSP

Women with MSP had significantly lower levels of vitamin D and lower daily calcium consumption compared to women without MSP (*P* < 0.001). A significantly higher proportion of women with MSP had vitamin D deficiency and insufficiency compared to women without MSP (95.4% vs. 76%; *P* < 0.001). Similarly, a significantly higher proportion of women with MSP had a calcium consumption of ≤450 mg/day, which is less than half of the recommended dietary allowance of 1,000–1,300 mg/day [31], compared to women without MSP (86.2% vs. 47.2%; *P* < 0.001). Regarding psychological symptoms, women with MSP had significantly higher HADS anxiety and depression scores compared to women without MSP (*P* < 0.001). The majority of women with MSP had abnormal and borderline abnormal HADS anxiety scores compared to women without MSP (74.1% vs. 19.4%; *P* < 0.001). Similarly, the majority of women with MSP had abnormal and borderline abnormal HADS depression scores compared to women without MSP (63.9% vs. 17.6%; *P* < 0.001) (Table 1).

### Prevalence and severity of premenstrual symptoms

Regarding premenstrual symptoms, the majority of participants experienced the physical and psychological symptoms of PMS. Fatigue was present in 87.5% of the entire study population (*n =* 189), breast tenderness in 74.1% (*n =* 160), and headache in 69.4% (*n =* 150). As far as psychological symptoms are concerned, mood swings were present in 87% (*n =* 188), anxiety in 76.9% (*n =* 166), irritability in 75% (*n =* 162), depression in 71.8% (*n =* 155), and social withdrawal in 66.7% (*n =* 144). Women with MSP had a significantly higher frequency of anxiety (83.3% vs. 68.5%; *P* = 0.02), depression (79.6% vs. 63.9%; *P* = 0.02), and social withdrawal (74.1% vs. 59.3%; *P* = 0.03) compared to women without MSP (Table 2).

**Table 2:** Prevalence and severity of selected premenstrual symptoms among the study population

Premenstrual symptoms	Women with MSP (*n* = 108)	Women without MSP (*n* = 108)	*P* value
**Fatigue**			
Yes	95 (88)	94 (87)	0.84
No	13 (12)	14 (13)	
**Fatigue severity**			
Absent	13 (12)	14 (13)	
Mild	18 (16.7)	44 (40.7)	<0.001^*^
Moderate	49 (45.4)	38 (35.2)	
Severe	28 (25.9)	12 (11.1)	
**Headache**			
Yes	82 (75.9)	68 (63)	0.05^*^
No	26 (24.1)	40 (37)	
**Headache severity**			
Absent	26 (24.1)	40 (37)	
Mild	28 (25.9)	43 (39.8)	<0.001^*^
Moderate	35 (32.4)	21 (19.4)	
Severe	19 (17.6)	4 (3.7)	
**Breast tenderness**			
Yes	80 (74.1)	80 (74.1)	1.00
No	28 (25.9)	28 (25.9)	
**Breast tenderness severity**			
Absent	29 (26.9)	28 (25.9)	
Mild	24 (22.2)	37 (34.3)	0.21
Moderate	37 (34.3)	31 (28.7)	
Severe	18 (16.7)	12 (11.1)	
**Irritability**			
Yes	86 (79.6)	76 (70.4)	0.12
No	22 (20.4)	32 (29.6)	
**Irritability severity**			
Absent	22 (20.4)	32 (29.6)	
Mild	14 (13)	40 (37)	<0.001^*^
Moderate	43 (39.8)	27 (25)	
Severe	29 (26.9)	9 (8.3)	
**Mood swings**			
Yes	99 (91.7)	89 (82.4)	0.07
No	9 (8.3)	19 (17.6)	
**Mood swings severity**			
Absent	9 (8.3)	19 (17.6)	
Mild	18 (16.7)	24 (22.2)	<0.01^*^
Moderate	38 (35.2)	48 (44.4)	
Severe	43 (39.8)	17 (15.7)	
**Anxiety**			
Yes	90 (83.3)	74 (68.5)	0.02^*^
No	18 (16.7)	34 (31.5)	
**Anxiety severity**			
Absent	18 (16.7)	34 (31.5)	
Mild	21 (19.4)	47 (43.5)	<0.001^*^
Moderate	41 (38)	19 (17.6)	
Severe	28 (25.4)	8 (7.4)	
**Depression**			
Yes	86 (79.6)	69 (63.9)	0.02^*^
No	22 (20.4)	39 (36.1)	
**Depression severity**			
Absent	22 (20.4)	39 (36.1)	
Mild	12 (11.1)	38 (35.2)	<0.001^*^
Moderate	42 (38.9)	23 (21.3)	
Severe	32 (29.6)	8 (7.4)	
**Social withdrawal**			
Yes	80 (74.1)	64 (59.3)	0.03^*^
No	28 (25.9)	44 (40.7)	
**Social withdrawal severity**			
Absent	28 (25.9)	44 (40.7)	<0.001^*^
Mild	17 (15.7)	38 (35.2)	
Moderate	38 (35.2)	21(19.4)	
Severe	25 (23.1)	5 (4.6)	

* Statistically significant. Data expressed as frequency (%).

Regarding the severity of physical and psychological premenstrual symptoms, women with MSP had a significantly higher frequency of moderate and severe symptoms of fatigue (71.3% vs. 46.3%; *P* < 0.001), headache (50% vs. 23.1%; *P* < 0.001), irritability (66.7% vs. 33.3%; *P* < 0.001), mood swings (75% vs. 60.1%; *P* = 0.001), anxiety (63.4% vs. 25%; *P* < 0.001), depression (68.5% vs. 28.7%; *P* < 0.001), and social withdrawal (58.9% vs. 24%; *P* < 0.001) compared to women without MSP (Table 2).

### Association between the severity of premenstrual symptoms and vitamin D status, daily calcium consumption, and HADS anxiety and depression scores

The chi-squared analysis of premenstrual symptom severity in relation to HADS anxiety, HADS depression, vitamin D status, and daily calcium consumption revealed that borderline and abnormal HADS anxiety and depression scores were significantly associated with a higher frequency of moderate-severe physical and psychological symptoms including fatigue, headache, breast tenderness, irritability, mood swings, anxiety, depression, and social withdrawal (*P* < 0.05; Table 3). Deficient and insufficient serum vitamin D levels were significantly associated with a higher frequency of moderate-severe headache, irritability, anxiety, and depression (*P* < 0.05). Low calcium consumption (<450 mg/day) was associated with a higher frequency of moderate or severe irritability, anxiety, depression, and social withdrawal (*P* < 0.05).

**Table 3:** Association between severity of premenstrual symptoms and other variables

Symptom	Variable	Symptom severity		*P* value
		Absent/mild	Moderate/severe	
Fatigue	**Serum vitamin D**			
	Normal (≥30 ng/dl) Insufficient (29–20 ng/dl) Deficient (<20 ng/dl)	15 (16.9) 16 (18) 58 (65.2)	16 (12.6) 16 (12.6) 95 (74.8)	0.32
	**Daily calcium consumption**			
	<230 mg/day 230–450 mg/day >450 mg/day	30 (33.7) 24 (27) 35 (39.3)	42 (33.1) 48 (37.8) 37 (29.1)	0.20
	**HADS anxiety score**			
	Normal (0–7) Borderline (8–10) Abnormal (11–21)	61 (68.5) 11 (12.4) 17 (19.1)	54 (42.5) 24 (18.9) 49 (38.6)	<0.01^*^
	**HADS depression score**			
	Normal (0–7) Borderline (8–10) Abnormal (11–21)	66 (74.2) 14 (15.7) 9 (10.1)	62 (48.8) 31 (24.4) 34 (26.8)	<0.01^*^
Headache	**Serum vitamin D**			
	Normal (≥30 ng/dl) Insufficient (29–20 ng/dl) Deficient (<20 ng/dl)	25 (18.2) 24 (17.5) 88 (64.2)	6 (7.6) 8 (10.1) 65 (82.3)	0.02^*^
	**Daily calcium consumption**			
	<230 mg/day	42 (30.7)	30 (38)	0.56
	230–450 mg/day	47 (34.3)	25 (31.6)	
	>450 mg/day	48 (35)	24 (30.4)	
	**HADS anxiety score**			
	Normal (0–7) Borderline (8–10) Abnormal (11–21)	92 (67.2)19 (13.9) 26 (19)	23 (29.1)16 (20.3) 40 (50.6)	<0.001^*^
	**HADS depression score**			
	Normal (0–7) Borderline (8–10) Abnormal (11–21)	103 (75.2) 19 (13.9) 15 (10.9)	25 (31.6) 26 (32.9) 28 (35.4)	<0.001^*^
Breast tenderness	**Serum vitamin D**			
	Normal (≥30 ng/dl) Insufficient (29–20 ng/dl) Deficient (<20 ng/dl)	19 (16.1) 20 (16.9) 79 (66.9)	12 (12.2) 12 (12.2) 74 (75.5)	0.39
	**Daily calcium consumption**			
	<230 mg/day 230–450 mg/day >450 mg/day	42 (35.6) 36 (30.5) 40 (33.9)	30 (30.6) 36 (36.7) 32 (32.7)	0.63
	**HADS anxiety score**			
	Normal (0–7) Borderline (8–10) Abnormal (11–21)	72 (61)20 (16.9) 26 (22)	43 (43.9)15 (15.3) 40 (40.8)	0.01^*^
	**HADS depression score**			
	Normal (0–7) Borderline (8–10) Abnormal (11–21)	78 (66.1) 23 (19.5) 17 (14.4)	50 (51) 22 (22.4) 26 (26.5)	<0.05^*^
Irritability	**Serum vitamin D**			
	Normal (≥30 ng/dl) Insufficient (29–20 ng/dl) Deficient (<20 ng/dl)	19 (17.6) 21 (19.4) 68 (63)	12 (11.1) 11 (10.2) 85 (78.7)	0.04^*^
	**Daily calcium consumption**			
	<230 mg/day 230–450 mg/day >450 mg/day	29 (26.9) 31 (28.7) 48 (44.4)	43 (39.8) 41 (38) 24 (22.2)	<0.01^*^
	**HADS anxiety score**			
	Normal (0–7) Borderline (8–10) Abnormal (11–21)	80 (74.1)16 (14.8) 12 (11.1)	35 (32.4)19 (17.6) 54 (50)	<0.001^*^
	**HADS depression score**			
	Normal (0–7) Borderline (8–10) Abnormal (11–21)	86 (79.6) 14 (13) 8 (7.4)	42 (38.9) 31 (28.7) 35 (32.4)	<0.001^*^
Mood swings	**Serum vitamin D**			
	Normal (≥30 ng/dl) Insufficient (29–20 ng/dl) Deficient (<20 ng/dl)	14 (20) 12 (17.1) 44 (62.9)	17 (11.6) 20 (13.7) 109 (74.7)	0.16
	**Daily calcium consumption**			
	<230 mg/day 230–450 mg/day >450 mg/day	17 (24.3) 28 (40) 25 (35.7)	55 (37.7) 44 (30.1) 47 (32.2)	0.14
	**HADS anxiety score**			
	Normal (0–7) Borderline (8–10) Abnormal (11–21)	52 (74.3) 7 (10) 11 (15.7)	63 (43.2) 28 (19.2) 55 (37.7)	<0.001^*^
	**HADS depression score**			
	Normal (0–7) Borderline (8–10) Abnormal (11–21)	55 (78.6) 7 (10) 8 (11.4)	73 (50) 38 (26) 35 (24)	<0.001^*^
Anxiety	**Serum vitamin D**			
	Normal (≥30 ng/dl) Insufficient (29–20 ng/dl) Deficient (<20 ng/dl)	22 (18.3) 21 (17.5) 77 (64.2)	9 (9.4) 11 (11.5) 76 (79.2)	<0.05^*^
	**Daily calcium consumption**			
	<230 mg/day 230–450 mg/day >450 mg/day	33 (27.5) 38 (31.7) 49 (40.8)	39 (40.6) 34 (35.4) 23 (24)	0.02^*^
	**HADS anxiety score**			<0.001^*^
	Normal (0–7) Borderline (8–10) Abnormal (11–21)	86 (71.7) 18 (15) 16 (13.3)	29 (30.2)17 (17.7) 50 (52.1)	
	**HADS depression score**			
	Normal (0–7) Borderline (8–10) Abnormal (11–21)	90 (75) 18 (15) 12 (10)	38 (39.6) 27 (28.1) 31 (32.3)	<0.001^*^
Depression	**Serum vitamin D**			
	Normal (≥30 ng/dl) Insufficient (29–20 ng/dl) Deficient (<20 ng/dl)	22 (19.8) 13 (11.7) 76 (68.5)	9 (8.6) 19 (18.1) 77 (73.3)	0.04^*^
	**Daily calcium consumption**			
	<230 mg/day 230–450 mg/day >450 mg/day	26 (23.4) 41 (36.9) 44 (39.6)	46 (43.8) 31 (29.5) 28 (26.7)	0.01^*^
	**HADS anxiety score**			
	Normal (0–7) Borderline (8–10) Abnormal (11–21)	82 (73.9) 14 (12.6) 15 (13.5)	33 (31.4) 21 (20) 51 (48.6)	<0.001^*^
	**HADS depression score**			
	Normal (0–7) Borderline (8–10) Abnormal (11–21)	89 (80.2) 13 (11.7) 9 (8.1)	39 (37.1) 32 (30.5) 34 (32.4)	<0.001^*^
Social withdrawal	**Serum vitamin D**			
	Normal (≥30 ng/dl) Insufficient (29–20 ng/dl) Deficient (<20 ng/dl)	22 (17.3) 22 (17.3) 83 (65.4)	9 (10.1) 10 (11.2) 70 (78.7)	0.11
	**Daily calcium consumption**			
	<230 mg/day 230–450 mg/day >450 mg/day	34 (26.8) 45 (35.4) 48 (37.8)	38 (42.7) 27 (30.3) 24 (27)	<0.05^*^
	**HADS anxiety score**			
	Normal (0–7) Borderline (8–10) Abnormal (11–21)	94 (74) 17 (13.4) 16 (12.6)	21 (23.6) 18 (20.2) 50 (56.2)	<0.001^*^
	**HADS depression score**			
	Normal (0–7) Borderline (8–10) Abnormal (11–21)	99 (78) 18 (14.2) 10 (7.9)	29 (32.6) 27 (30.3) 33 (37.1)	<0.001^*^

* Statistically significant. Data expressed as frequency (%).

### Association between daily calcium consumption, serum vitamin D levels, and psychological symptoms

We also examined the relationships between daily calcium consumption, serum vitamin D levels, and HADS subclass scores for anxiety and depression. We observed significant associations between daily calcium consumption and the HADS subclass score for anxiety (*P* < 0.001) and depression (*P* = 0.001), a higher daily calcium consumption being associated with a lower frequency of abnormal HADS anxiety and depression scores. Similarly, we observed significant associations between serum vitamin D levels and the HADS subclass score for anxiety (*P* < 0.001) and depression (*P* = 0.02), sufficient vitamin D levels being associated with a lower frequency of abnormal HADS anxiety and depression scores (Table 4).

**Table 4:** Association between daily calcium consumption, serum vitamin D, and psychological symptoms

Variables	Daily calcium consumption			*P* value
	1^st^ tertile (<230 mg/day)	2^nd^ tertile (230–450 mg/day)	3^rd^ tertile (≥450 mg/day)	
**HADS anxiety score**				
Normal (0–7)	26 (36.1)	36 (50)	53 (73.6)	<0.001^*^
Borderline (8–10)	12 (16.7)	15 (20.8)	8 (11.1)	
Abnormal (11–21)	34 (47.2)	21 (29.2)	11 (15.3)	
**HADS depression score**				0.001^*^
Normal (0–7)	31 (43.1)	44 (61.1)	53 (73.6)	
Borderline (8–10)	19 (26.4)	12 (16.7)	14 (19.4)	
Abnormal (11–21)	22 (30.6)	16 (22.2)	5 (6.9)	
**Variables**	**Serum vitamin D**			***P* value**
	**Normal** **(≥30 ng/dl)**	**Insufficient** **(29–20 ng/dl)**	**Deficient** **(<20 ng/dl)**	
**HADS anxiety score**				<0.001^*^
Normal (0–7)	25 (80.6)	24 (75)	66 (43.1)	
Borderline (8–10)	1 (3.2)	2 (6.2)	32 (20.9)	
Abnormal (11–21)	5 (16.1)	6 (18.8)	55 (35.9)	
**HADS depression score**				0.02^*^
Normal (0–7)	26 (83.9)	19 (59.4)	83 (54.2)	
Borderline (8–10)	3 (9.7)	9 (28.1)	33 (21.6)	
Abnormal (11–21)	2 (6.5)	4 (12.5)	37 (24.2)	

* Statistically significant. Data were expressed as frequency (%).

## DISCUSSION

This study compared the frequency and severity of premenstrual symptoms in relation to vitamin D status, daily calcium consumption, and psychological symptoms among women with and without MSP.

We observed a high prevalence of vitamin D deficiency and insufficiency, a high prevalence of borderline and abnormal HADS anxiety and depression scores, and a high prevalence of low daily calcium consumption among women with MSP compared to women without MSP.

Women with MSP had a higher prevalence of moderate and severe physical and psychological premenstrual symptoms including fatigue, headache, irritability, mood swings, anxiety, depression, and social withdrawal compared to women without MSP. More severe premenstrual symptoms were significantly associated with abnormal HADS anxiety and depression scores. Furthermore, increased severity of headache, irritability, anxiety, and depression was significantly associated with deficient serum vitamin D levels, and increased severity of irritability, anxiety, depression, and social withdrawal were significantly associated with low daily calcium consumption. Abnormal HADS anxiety and depression scores were significantly associated with deficient vitamin D levels and low daily calcium consumption.

Vitamin D is a fat-soluble vitamin that has critical roles in muscle and brain functions, with receptors expressed in these tissues [32,33]. An association between vitamin D deficiency, MSP, and psychological symptoms was previously reported among patients with MSP [26,27,34–36]. In accordance with these findings, we have documented a high prevalence of vitamin D deficiency, as well as borderline and abnormal HADS anxiety and depression scores among women with MSP. Deficient vitamin D levels were significantly associated with abnormal psychological symptoms.

In this study, women with MSP had a significantly higher prevalence of severe physical and psychological premenstrual symptoms than women without MSP, and the severity of premenstrual symptoms was significantly associated with abnormal psychological symptoms. These findings suggest an association between vitamin D deficiency, pain, anxiety, depression, and the severity of premenstrual symptoms, and are in agreement with those of Firoozi *et al*. [9], who reported that more severe PMS is associated with an increase in the mean scores of psychiatric symptoms, including anxiety and depression, and interpersonal sensitivity.

Similarly to vitamin D, calcium has an important role in the central nervous system, and it may have crucial implications in the etiology of many neuropsychiatric disorders [37–39]. In this study, women with MSP had significantly lower daily calcium consumption than women without MSP. We found a significant association between low calcium consumption and abnormal HADS anxiety and depression scores, suggesting a relation between daily calcium consumption and the risk of psychological symptoms, which significantly exacerbate premenstrual symptoms. Our results are consistent with those of Del Mar Fernández *et al*. [40], Firoozi *et al*. [9], and other cross-sectional studies [11,13,16,41] that documented that psychological factors are related to the severity of PMS. In addition, similar findings were reported in a study conducted by Bae and Kim [42], in which a negative association was found between self-rated depression scores and daily calcium consumption among middle-aged women. Interestingly, supplementation of a multivitamin combination containing calcium, magnesium, and zinc has been found to reduce anxiety and perceived stress in 80 healthy male volunteers [43].

The findings of this study regarding the association between vitamin D, daily calcium consumption, and premenstrual symptoms are similar to those of a systematic review that showed that low serum levels of calcium and vitamin D during the luteal phase of the menstrual cycle caused or exacerbated the symptoms of PMS [44]. However, other systematic reviews have found no significant association between these factors [45].

In previous studies, the supplementation of vitamin D and calcium resulted in a significant reduction in the severity of MSP and psychological symptoms among patients with MSP [26], psychiatric outpatients [27], and patients with overactive bladder [35]. Similar findings were observed by Wepner *et al*. [46], Le Goaziou *et al*. [47], and Muir and Montero-Odasso [48], suggesting that interventional studies are needed to assess whether vitamin D and calcium supplements can reduce the severity of PMS symptoms among women with MSP.

A systematic review conducted by Arab *et al*. found that in most of the included studies serum calcium levels were lower in women with PMS, and that calcium supplementation was beneficial in decreasing the incidence of PMS and its related symptoms [49].

The findings of our study suggest that MSP could be implicated in increasing the severity of premenstrual symptoms. MSP itself could be caused by low vitamin D levels and low daily calcium consumption, which could also be implicated in increasing the severity of psychological symptoms including anxiety and depression. Therefore, MSP, low vitamin D levels, low daily calcium consumption, psychological symptoms, and the severity of premenstrual symptoms may be interrelated and may exacerbate each other.

This study is one of the first to investigate the severity of premenstrual symptoms in relation to serum vitamin D levels, daily dietary calcium consumption, and psychological symptoms in women with MSP. The case–control study design enabled us to compare the levels of our variables between the two study groups, and the sample size was adequate to demonstrate significant differences in many variables.

The main limitation of our study is that we did not assess the effect of vitamin D and calcium supplements on reducing MSP and the severity of premenstrual symptoms, and further studies are needed to investigate these aspects.

## CONCLUSION

This study revealed a high prevalence of vitamin D deficiency, abnormal psychological symptoms, low daily calcium consumption, and high frequency of moderate and severe physical and psychological premenstrual symptoms among women with MSP compared to women without MSP. Abnormal HADS scores for anxiety and depression were associated with increased severity of premenstrual symptoms. Deficient vitamin D levels and low daily calcium consumption were associated with increased severity of premenstrual headache, irritability, anxiety, and depression. Low daily calcium consumption was associated with increased severity of premenstrual irritability, anxiety, depression, and social withdrawal. These results suggest that vitamin D deficiency, low calcium consumption, psychological symptoms, and MSP could be interrelated and implicated in increasing the severity of premenstrual symptoms.

## Data Availability

Further data is available from the corresponding author upon reasonable request.
